# The Influence of Kinetic Models and Attenuation Correction on Cadmium–Zinc–Telluride Single-Photon Emission Computed Tomography (CZT SPECT)-Derived Myocardial Blood Flow and Reserve: Correlation with Invasive Angiography Data

**DOI:** 10.3390/jcm13051271

**Published:** 2024-02-23

**Authors:** Andrey Mochula, Alina Maltseva, Kristina Kopeva, Elena Grakova, Olga Mochula, Konstantin Zavadovsky

**Affiliations:** 1 Nuclear Department, Cardiology Research Institute, Tomsk National Research Medical Center, Russian Academy of Sciences, Tomsk 634012, Russia; mochula.andrew@gmail.com (A.M.); maltseva.alina.93@gmail.com (A.M.); konstzav@gmail.com (K.Z.); 2 Department of Myocardial Pathology, Cardiology Research Institute, Tomsk National Research Medical Center, Russian Academy of Sciences, Tomsk 634012, Russia; gev@cardio-tomsk.ru; 3 Department of Radiology and Tomography, Cardiology Research Institute, Tomsk National Research Medical Center, Russian Academy of Sciences, Tomsk 634012, Russia; mochula.olga@gmail.com

**Keywords:** coronary flow reserve, myocardial blood flow, kinetic models, attenuation correction, dynamic SPECT

## Abstract

(1) **Background**: The objective of this study was to determine the optimal post-processing model for dynamic cadmium–zinc–telluride single-photon emission computed tomography (CZT-SPECT). (2) **Methods**: A total of 235 patients who underwent diagnostic invasive coronary angiography within three months of the SPECT and those who had coronary computed tomography angiography (CCTA) before SPECT (within 3 months) were enrolled in this study. Each SPECT study was processed to obtain global and regional stress myocardial blood flow (sMBF), rest-MBF (rMBF), myocardial flow reserve (MFR) and flow difference (FD) estimates obtained with 1-tissue-compartment (1TCM) and net retention (NR) modes, both with and without attenuation correction. (3) **Results**: The use of AC led to significantly higher sMBF, rMBF and DF values obtained by 1TCM compared those values derived by 1TCM with NAC; the lowest values of stress MBF and rest MBF were obtained by 1TCM_NAC. The resting flow, MFR and DF were significantly (*p* < 0.005) higher in the AC model than in NAC. All quantitative variables were significantly (*p* < 0.05) higher in NR_NAC than in the 1TC_NAC model. Finally, sMBF, rMBF and FD showed significantly (*p* < 0.05) higher values by using 1TMC_AC compared to NR_AC. (4) **Conclusions**: We suggested that 1-compartment and net retention models correctly reflect coronary microcirculation and can be used for clinical practice for evaluating quantitative myocardial perfusion by dynamic SPECT. Attenuation correction is an important step in post-processing dynamic SPECT data, which increases the consistency and diagnostic accuracy of models.

## 1. Introduction

The quantitative assessment of myocardial perfusion is a superior method to evaluate micro- and macrocirculation in the myocardium. This method is based on the evaluation of myocardial blood flow (MBF) and coronary flow reserve (CFR) values. For a long time, positron emissions tomography (PET) with 15O, 13N and 82Rb was the only noninvasive approach for assessing MBF and CFR [1,2]. Nowadays, the quantitative indices of myocardial blood flow can be assessed by single-photon emission computed tomography (SPECT) [3,4]. Cadmium–zinc–telluride (CZT)-based gamma cameras allow one to perform dynamic tomographic first-pass imaging data analysis of the tracer through the left-side chambers. As a result, regional and global quantitative parameters can be evaluated by dynamic CZT-SPECT.

The vast majority of research in this area focuses on feasibility evaluation, validation and diagnostic efficiency assessment of this method. The latest investigations showed that there is good correlation between dynamic CZT-SPECT and PET or fractional flow reserve (FFR) methods [4].

However, there is a lack of studies focused on the standardization of dynamic CZT-SPECT. At the moment, different research groups are using various types of dynamic SPECT protocols, stress tests and post-processing [4,5,6,7,8]. There are data showing the need to use motion correction; however, the impact of attenuation correction has not been proven yet. Additionally, the influence of the post-processing flow models on the diagnostic accuracy of dynamic CZT-SPECT has not been evaluated.

In this study, we attempted to determine the optimal post-processing model for dynamic CZT-SPECT.

## 2. Materials and Methods

### 2.1. Patient Population and Study Design

**Figure 1:**
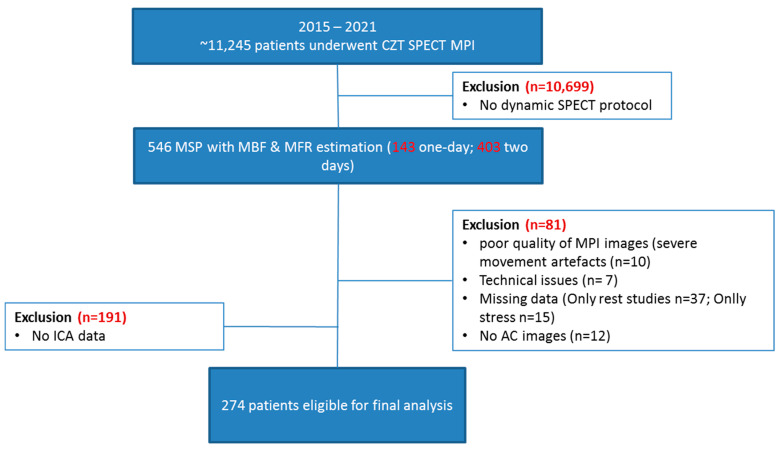
Flowchart diagram.

From September 2015 to April 2021, a total of 11,245 stable consecutive patients hospitalized at Cardiology Research Institute, Tomsk National Research Medical Center of Russian Academy of Science, were referred to myocardial perfusion scintigraphy according to clinical indications. Patients (*n* = 546) who underwent diagnostic invasive coronary angiography (ICA) within three months of SPECT and those who had coronary computed tomography angiography (CCTA) before SPECT (within 3 months) were included in the final analysis (Figure 1). Each study was processed to obtain global and regional stress myocardial blood flow (sMBF), rest-MBF (rMBF), myocardial flow reserve (MFR) and flow difference (FD) values by using 1-tissue-compartment (1TCM) and net retention (NR) modes, both with and without attenuation correction, which served as primary endpoints.

The exclusion criteria were previous coronary artery bypass grafting, severe bronchial asthma and/or chronic obstructive pulmonary disease in the acute phase; advanced heart failure; moderate or severe stenosis and/or insufficiency of the heart valves; significant obstruction of LV; prior pulmonary embolism with pulmonary hypertension ≥45 mmHg; acute and chronic inflammatory heart disease; ventricular extrasystole ≥3 grades (Lown); atrioventricular block II-III degree and/or sinus node weakness syndrome; significant obstruction of the left ventricular outflow tract; contraindication to adenosine or iodine contrast administration. This set of exclusion criteria was selected to avoid adenosine stress-test-related complications.

Moreover, patients with cardiac interventions or medical therapy optimization between dynamic SPECT and ICA or CCTA and SPECT were excluded from the analysis to minimize the possible inconsistency between dynamic SPECT and coronary angiography results.

### 2.2. Coronary Angiography and Fractional Flow Reserve

Quantitative coronary arteriography was performed on Axiom Artis coronary angiography system (Siemens; Erlangen, Germany). The protocol of ICA and fractional flow reserve (FFR) was previously published [9]. Briefly, all coronary artery stenoses were quantitatively assessed using dedicated software by an experienced reader. FFR assessment was performed using ILUMIEN console (St. Jude Medical, St. Paul, MN, USA) and 0.014 pressure-monitoring Wire Aeris (St. Jude Medical) in patients with intermediate (40–90%) coronary artery stenosis. Maximal hyperemia was obtained by intravenous (i.v.) infusion of adenosine triphosphate (ATP). The FFR values ≤ 0.80 were deemed indicative of significant inducible ischemia.

### 2.3. CZT SPECT Imaging

Patients were instructed to refrain from caffeine and methylxanthine-containing substances and to avoid nitrates, calcium channel blockers, and beta-blockers for at least 24 h before the scan. All scans were performed after overnight fasting.

CZT-SPECT was performed on Discovery NM/CT 570c (GE Healthcare, Haifa, Israel) according to rest–stress single-day or two-day protocol. Low-dose CT scan was performed before the first dynamic acquisition for attenuation correction (AC) and heart positioning. In the one-day protocol, rest acquisition was performed first with 3 MBq/kg and stress with 9 MBq/kg, while in two-day protocol, 5 MBq/kg was used for both rest and stress studies. 99mTc-Sestamibi was injected using a syringe pump intravenously as a 5 mL bolus (injection rate 1 mL/s) followed by saline flush (30 mL with the rate 2 mL/s, using an automatic injector Ulrich Missouri XD 2001 Ulrich GmbH & Co. KG, Ulm Germany). List-mode ECG-gated dynamic data acquisition started 10 sec prior to the 99mTc-Sestamibi injection and acquired for 610 s. After 40 min from tracer injection, a 7 min long standard ECG-gated (16 framed per cardiac cycle) rest acquisition was performed using a dedicated patient positioning application in order to obtain the same coordinates of the heart as for the previous scan [9,10,11].

After 2 min of intravenous infusion of adenosine (160 mcg/kg/min), the second dose of 99mTc Sestamibi (9 MBq/kg) was injected, and list-mode dynamic data acquisition of 610 s was started 10 s prior to the radiotracer injection. The infusion of adenosine continued for an additional 2 min [12]. After that, as for the rest scan, patients were removed from the gamma camera, and a stress standard ECG-gated scan was acquired after 45 min from the tracer injection.

Dosimetry. The mean 99mTc-MIBI dose at rest was 258.9 ± 42.9 MBq (range 192–390 MBq); at stress 776.9 ± 128.7 (range 576–1170 MBq). The low-dose CT scan dose was 0.27 mSv. The mean effective radiation dose was 8.46 ± 1.37 mSv (range 6.41–12.75 mSv) per patient.

### 2.4. CZT SPECT Analysis

#### 2.4.1. Conventional MPI Image Processing and Analysis

Low-dose CT scans were transferred to Xeleris workstation to obtain AC maps. The alignment of perfusion and CT data was performed with a visual control. Images were reconstructed on the dedicated workstation (Xeleris 4.0; GE Healthcare, Haifa, Israel) using maximum-penalized-likelihood iterative reconstruction (60 iterations; Green OSL Alpha 0.7; Green OSL Beta 0.3). The software Myovation for Alcyone (GE Healthcare, Haifa, Israel) was used for image reconstruction, and Butterworth post-processing filter (frequency 0.37; order 7) was applied to the reconstructed slices. The reconstruction was performed in 70 × 70 pixel matrix with 50 slices. Raw MPS-CZT data at stress and at rest were visually analyzed for motion and attenuation artefacts. Stress/rest images were analyzed with a commercially available software package, Corridor 4DM (University of Michigan, Ann Arbor, MI, USA), on AC images. Each of 17 segments was scored based on semi-quantitative 5-point scoring system (from 0 = normal uptake to 4 = absent radiotracer distribution) [11,13]. Accordingly, the sum of the stress scores of all segments (SSS) and the sum of the rest scores of all segments (SRS) were quantified. A summed difference score (SDS) was calculated as the difference between SSS and SRS. Image processing was performed at the Core Facility “Medical Genomics” (Tomsk National Research Medical Centre, Tomsk, Russia).

#### 2.4.2. Dynamic SPECT Image Processing and Analysis

Dynamic CZT imaging was processed as previously published [9]. In brief, each rest and stress list-file datum was initially reconstructed (in 70 × 70 pixels matrix; 50 slices) and re-binned into 20 frames: 12 frames of 10 s each and 8 frames of 30 s each. In cases of one-day rest–stress protocol, the residual activity was extracted using CFR “CrossTalk” application to obtain correct absolute MBF values on the stress dataset.

The reframed and corrected (for one day protocol) dynamic images were reconstructed using penalized maximum-likelihood expectation maximization iterative algorithm (60 iterations; regularization type: OSL green; Green OSL Alpha 0.7, Green OSL Beta 0.5). Finally, the reconstructed dynamic images as well as CT attenuation correction maps were processed by «4DM SPECT CFR» v.2017 (INVIA, Ann Arbor, MI, USA). The region of interest (ROI) for input function was located on the mitral valve plane, including parts of LV cavity and left atrium. The size of this ROI was 4 mm on short-axis view and 30 mm on long-axis views. Manual motion correction of dynamic dataset was performed in accordance with manufacturer’s recommendation [11]. Each study was processed by two independent experienced readers (AVM, KWZ). Discrepancies were resolved by consensus. Time–activity curves for the whole left ventricular myocardium as well as for the left anterior descending coronary artery (LAD), left circumflex coronary artery (LCX), and right coronary artery (RCA) vessel territories were generated semi-automatically. The myocardial uptake (K1) and retention rate (R) were estimated with and without AC using 1-tissue-compartment kinetic model and net retention (NR) model, respectively [14,15]. To convert the tracer uptake and retention rate to MBF values, the Renkin–Crone flow model was used. The following flow model parameters were used: 1) 1TCM_AC α = 0.879, β = 0.337; (2) 1TCM_NAC α = 0.814, β = 0.473; NetRet_AC α = 0.880, β = 0.208; NetRet_NAC α = 0.860, β = 0.290. The value of MFR was calculated as MBF ratio (MBF stress/MBF rest). Additionally, the absolute difference between stress MBF and rest MBF as flow difference (FD) was calculated.

### 2.5. Statistical Analysis

Statistical analyses were performed using STATISTICA 10.0; Jamovi v. 2.2.5. Shapiro–Wilk analysis was provided for assessment of data distribution. Continuous values are given as Me (Q1–Q3). Categorical variables are described as number followed by percentage in parentheses. Mann–Whitney, ANOVA Kruskal–Wallis + post hoc Tukey test, and Fisher’s exact test were used for comparison of continuous values and categorical variables. Spearman’s test was used to estimate the correlation coefficient between quantitative variables. Categorical variables were compared using Fisher’s exact test. Bland–Altman analysis was used for assessment of the agreement between models. All *p*-values are two-tailed. In all statistical analyses, a two-tailed *p*-value of 0.05 or less was considered to indicate statistical significance.

## 3. Results

### 3.1. Study Population

A flowchart of the study is presented in Figure 1. A total of 235 patients were enrolled in this study. The detailed clinical characteristics of patients are presented in Table 1. Most patients were male (71%), more than one-half had hypercholesterolemia, and more than two-thirds had hypertension. Diabetes milieus was diagnosed in 28% of cases. In 74% of cases, coronary artery disease (CAD) was known, and 78% patients had chest pain. Less than half of patients were current smokers; body mass index was over 30 in 43% of cases.

### 3.2. ICA and CCTA Results

A total of 181 patients underwent ICA after the SPECT study, and in 54 patients, previous CCTA data were used for the analysis. Detailed ICA data are presented in Table 2. According to the patient-based analysis, 164 (70%) patients had obstructive and 71 (30%) nonobstructive CAD. According to vessel-based analysis, a total of 705 vessels were analyzed, and 335 (50%) were considered as having an obstruction lesion. No LM alone stenosis >50% was revealed.

Obstructive lesions were mainly located in LAD. A total of 26 patients underwent FFR assessment, and a total of 13 (37%) vessels with FFR ≤ 0.8 were reviewed, mainly in LAD (77%).

### 3.3. SPECT Results

The results of the semi-quantitative MPS parameters are presented in Table 1. A total of 104 (44%) patients had normal myocardial perfusion (SSS < 4); 65 (27.5%), 35 (15%), and 31 (13.5%) patients had small, moderate, and large perfusion defects, respectively. A total of 193 (82%) had a normal (>50%) LV ejection fraction, whereas 9 (4%) and 33 (14%) had mild ranging and reduced LVEF, respectively [15,16].

### 3.4. Dynamic SPECT Results

Table 3 summarizes the CZT SPECT-derived global quantitative values, estimated by using 1TCM and NRM, with and without AC. The use of AC led to significantly higher sMBF, rMBF, and DF values obtained by 1TCM, as compared to values derived by 1TCM with NAC; the lowest values of stress MBF and rest MBF were obtained by 1TCM_NAC. The resting flow, MFR, and DF were significantly (*p* < 0.005) higher with AC, as compared to NAC. All quantitative variables were significantly (*p* < 0.05) higher by NR_NAC, as compared to 1TC_NAC. Finally, sMBF, rMBF, and FD showed significantly (*p* < 0.05) higher values by using 1TMC_AC compared to NR_AC.

### 3.5. Agreement between Flow Models

Correlation analysis showed a strong relationship among four models for stress and rest MBF, MFR, and DF (Supplementary Tables S1–S4, Supplementary Figure S1).

According to the Bland–Altman analysis, absolute values of stress MBF showed no agreement between different models, regardless of the attenuation correction (Table 4). The values of rMBF also showed no agreements, except for NR_AC vs. NR NAC. The biases gradually increased with the size of the variables. In contrast to the absolute values, MFR showed agreement between models with AC but poor agreement with NAC. The values of FD showed no agreement, except 1TCM_AC vs. 1TCM_NAC.

Bland–Altman plots are presented in Supplementary Figure S3.

### 3.6. Comparison with Coronary Angiography Data

According to patient-based analysis (Table 5), all global values of sMBF, MFR, and DF were significantly (*p* < 0.05) lower in patient samples with obstructive CAD compared to the nonobstructive one. Rest MBF values showed significant differences, except for the NR_AC model. Generally, flow models with AC demonstrated higher differences between dynamic SPECT indices, as compared to NAC, except for rest MBF values by 1TCM_NAC.

Vessel-based analysis (Table 6) also revealed significantly (*p* < 0.001) decreased regional values of sMBF, MFR, and FR estimated by 1TCM and NRM, with AC and with NAC in vessels with obstructive plaque compared to those with no obstructive lesion. Stress MBF, MFR, and DF derived by the AC models were lower in comparison to NAC data. Rest MBF was significantly (*p* < 0.05) lower for NR_AC. However, it was not correct for stress MBF.

**Table 3:** Model comparison.

Flow Model					Kruskal-Wallis H Test *p* Value	Intergroup Comparison, *p*			
Global Value	1TCM_AC	1TCM_NAC	NR_AC	NR_NAC		1TCM_AC vs. 1TCM_NAC	NR_AC vs. NR_NAC	1TCM_AC vs. NR_AC	1TCM_NAC vs. NR_NAC
Stress MBF	1.46 (0.94; 1.95)	0.97 (0.63; 1.41)	1.17 (0.73; 1.59)	1.33 (0.75; 1.82)	<0.001	* 10.45; <0.001	1.0; 0.318	* 6.68; <0.001	* 4.77; <0.001
Rest MBF	0.76 (0.55; 1.12)	0.49 (0.37; 0.64)	0.55 (0.41; 0.81)	0.56 (0.37; 0.83)	<0.001	* 12.99; <0.001	* 1.59; 0.0113	* 8.16; <0.001	* 3.25; 0.001
MFR	1.74 (1.21; 2.61)	1.82 (1.40; 2.51)	1.93 (1.31; 2.61)	2.13 (1.56; 2.76)	<0.001	1.83; 0.068	* 2.56; 0.01	* 2.02; 0.04	* 2.74; 0.006
FD	0.56 (0.16; 1.22)	0.45 (0.20; 0.81)	0.48 (0.19; 0.91)	0.64 (0.30; 1.09)	<0.001	* 3.16; 0.002	* 3.77; <0.001	* 1.98; 0.049	* 4.96; <0.001

Note. *p*—statistical significance (*—*p* < 0.05); 1TCM—one tissue compartment mode; AC—attenuation correction; NAC—non-attenuation correction; FD—flow difference; MBF—myocardial blood flow; MFR—myocardial flow reserve.

**Table 4:** Bland–Altman model comparison analysis.

Flow Model					Bland–Altman Analysis (bias (95% CI), *p*)			
Global Value	1TCM_AC	1TCM_NAC	NR_AC	NR_NAC	1TCM_AC vs. 1TCM_NAC	NR_AC vs. NR_NAC	1TCM_AC vs. NR_AC	1TCM_NAC vs. NR_NAC
Stress MBF	1.46 (0.94; 1.95)	0.97 (0.63; 1.41)	1.17 (0.73; 1.59)	1.33 (0.75; 1.82)	* 0.49 (0.39/0.59), <0.0001	* −0.09 (−0.18/−0.016), 0.02	* 0.36 (0.29/0.43), <0.0001	* −0.2 (−0.31/−0.15), <0.0001
Rest MBF	0.76 (0.55; 1.12)	0.49 (0.37; 0.64)	0.55 (0.41; 0.81)	0.56 (0.37; 0.83)	* 0.37 (0.31/0.44), <0.0001	0.03 (−0.01/0.07), 0.17	* 0.27 (0.22/0.32), <0.0001	* −0.08 (−0.11/−0.03), <0.0001
MFR	1.74 (1.21; 2.61)	1.82 (1.40; 2.51)	1.93 (1.31; 2.61)	2.13 (1.56; 2.76)	0.03 (−0.1/0.2), 0.6	−0.13 (−0.25/0.0009), 0.05	−0.09 (−0.19/0.02), 0.11	* −0.24 (−0.34/−0.2), <0.0001
FD	0.56 (0.16; 1.22)	0.45 (0.20; 0.81)	0.48 (0.19; 0.91)	0.64 (0.30; 1.09)	0.11 (−0.002/0.22), 0.05	* −0.14 (−0.21/−0.06), 0.0006	* 0.09 (0.022/0.16), 0.01	* −0.15 (−0.21/−0.09), <0.0001

Note. *p*—statistical significance (*—*p* < 0.05); CI—confidence interval; 1TCM—one tissue compartment mode; AC—attenuation correction; NAC—non-attenuation correction; FD—flow difference; MBF—myocardial blood flow; MFR—myocardial flow reserve.

**Table 5:** Global quantitative CZT SPECT values in obstructive and nonconstructive CAD patients (patient-based analysis).

Global Values	Obstructive CAD (ICA) *n* = 164				Nonobstructive CAD (ICA) *n* = 71				Mann–Whitney Test			
	1TCM_AC	1TCM_NAC	NR_AC	NR_NAC	1TCM_AC	1TCM_NAC	NR_AC	NR_NAC	*p* * 1 vs. 5	*p* 2 vs. 6	*p* 3 vs. 7	*p* 4 vs. 8
	1	2	3	4	5	6	7	8				
Stress MBF	1.27 (0.79; 1.92)	0.86 (0.51; 1.24)	1.08 (0.61; 1.55)	1.15 (0.50; 1.91)	1.64 (1.27; 2.29)	1.06 (0.77; 1.55)	1.33 (1.01; 1.77)	1.42 (1.00; 1.79)	0.002 −29% *	0.0015 −23% *	0.00095 −23% *	0.029 −23.5% *
Rest MBF	0.75 (0.55; 1.14)	0.47 (0.34; 0.61)	0.53 (0.37; 0.80)	0.55 (0.28; 0.85)	0.81 (0.55; 1.08)	0.53 (0.40; 0.71)	0.56 (0.44; 0.85)	0.56 (0.43; 0.71)	0.83 −8%	0.04 −12.8% *	0,14 −5.7%	0.36 −1.8%
MFR	1.59 (1.05; 2.38)	1.64 (1.21; 2.43)	1.75 (1.19; 2.59)	2.01 (1.36; 2.61)	2.14 (1.46; 2.78)	1.99 (1.65; 2.68)	2.23 (1.46; 2.74)	2.31 (1.83; 3.07)	0.0004 −34.6% *	0.002 −21.3% *	0.027 −27.4% *	0.002 −15%
FD	0.40 (0.02; 1.15)	0.33 (0.14; 0.70)	0.36 (0.12; 0.85)	0.48 (0.17; 1.13)	0.84 (0.40; 1.30)	0.64 (0.31; 0.94)	0.71 (0.33; 1.08)	0.82 (0.49; 1.08)	0.0006 −110% *	0.004 −93.9% *	0.0015 −97% *	0.012 −70.8% *

Note. *p*—statistical significance (*—*p* < 0.05); 1TCM—one tissue compartment mode; AC—attenuation correction; NAC—non-attenuation correction; FD—flow difference; MBF—myocardial blood flow; MFR—myocardial flow reserve.

**Table 6:** Global quantitative CZT SPECT values in obstructive and nonconstructive CAD patients (vessel-based analysis).

	Obstructed Vessel (*n* = 355)				Nonobstructed Vessel (*n* = 350)				Mann–Whitney Test			
Regional Values	1TCM_AC	1TCM_NAC	NR_AC	NR_NAC	1TCM_AC	1TCM_NAC	NR_AC	NR_NAC	*p* * 1 vs. 5	*p* 2 vs. 6	*p* 3 vs. 7	*p* 4 vs. 8
	1	2	3	4	5	6	7	8				
Stress MBF	1.37 (0.89; 2.06)	0.84 (0.57; 1.25)	0.98 (0.62; 1.45)	1.01 (0.49; 1.68)	1.80 (1.25; 2.56)	1.19 (0.78; 1.78)	1.36 (0.98; 1.82)	1.39 (0.93; 1.89)	0.000000 −31.4% *	0.000000 −41.7% *	0.000000 −38.8% *	0.000003 −37.6% *
Rest MBF	0.94 (0.65; 1.54)	0.58 (0.37; 0.76)	0.57 (0.39; 0.95)	0.57 (0.28; 0.92)	0.98 (0.64; 1.39)	0.59 (0.42; 0.82)	0.63 (0.46; 0.96)	0.61 (0.42; 0.81)	0.928578 −4.2%	0.058833 −1.7%	0.013796 −10.5% *	0.074727 −7%
MFR	1.43 (0.92; 2.10)	1.56 (1.16; 2.14)	1.51 (1.08; 2.29)	1.75 (1.27; 2.34)	1.81 (1.26; 2.60)	1.96 (1.50; 2.63)	2.01 (1.44; 2.71)	2.21 (1.67; 2.69)	0.000000 −26.5% *	0.000000 −25.6% *	0.000001 −33% *	0.000000 −26% *
FD	0.31 (−0.12; 0.83)	0.29 (0.08; 0.61)	0.30 (0.03; 0.67)	0.39 (0.09; 0.90)	0.78 (0.32; 1.29)	0.61 (0.27; 1.07)	0.67 (0.26; 0.99)	0.75 (0.43; 1.11)	0.000000 −151.6% *	0.000000 −110% *	0.000000 −123% *	0.000000 −92% *

Note. *p*—statistical significance (*—*p* < 0.05); 1TCM—one tissue compartment mode; AC—attenuation correction; NAC—non-attenuation correction; FD—flow difference; MBF—myocardial blood flow; MFR—myocardial flow reserve.

ROC analysis was performed for the diagnostic accuracy assessment of the models in identifying obstructive lesions (Table 7).

The area under the curve (AUC) for regional stress MBF, MFR, and DF was close for all models. Noticeably, models with AC showed higher sensitivity, whereas the NAC ones had better specificity. Also, the cut-off values were close for all models.

### 3.7. Comparison with FFR

FFR data were acquired from 35 vessel areas in 26 patients. According to the FFR results, patients were divided into two subgroups, with FFR ≤ 0.8 (*n* = 13) and FFR > 0.8 (*n* = 22).

Regional values of stress MBF, CFR, and DF obtained by NR_AC and 1TC_AC were lower (*p* < 0.05) in patients with hemodynamically significant coronary artery stenosis. Resting myocardial blood flow was not significantly different (Table 8). Based on the ROC analysis, neither stress MBF nor CFR and DF showed statistical significance in the identification of the hemodynamic significant stenosis 1TC_NAC model.

The MFR acquired by the NR_AC and 1TC_AC models demonstrated more accurate values of sensitivity, specificity, and higher AUC (Table 9, Supplementary Figure S2).

**Table 8:** Vessel-based analysis (FFR).

Regional Values	FFR ≤ 0.8 (*n* = 13)				Normal FFR (*n* = 22)				Mann–Whitney Test			
	1TCM_AC	1TCM_NAC	NR_AC	NR_NAC	1TCM_AC	1TCM_NAC	NR_AC	NR_NAC	*p* * 1 vs. 5	*p* 2 vs. 6	*p* 3 vs. 7	*p* 4 vs. 8
	1	2	3	4	5	6	7	8				
sMBF	1.99 (1.33; 2.52)	1.79 (1.08; 2.10)	1.11 (0.99; 1.59)	1.98 (1.42; 2.33)	2.07 (1.62; 3.44)	2.12 (1.42; 2.75)	1.91 (1.52; 2.25)	2.44 (1.98; 3.15)	0.229010 +4%	0.156032 +18.4%	0.001540 +72% *	0.033271 +23% *
rMBF	1.37 (0.84; 2.31)	0.75 (0.51; 0.89)	0.87 (0.68; 1.20)	0.90 (0.70; 1.23)	1.07 (0.70; 2.42)	0.69 (0.43; 0.78)	0.87 (0.47; 1.11)	0.94 (0.77; 1.09)	0.532807 −22%	0.631643 −8%	0.489284 −0%	0.889406 +4.4%
MFR	1.09 (0.91; 1.76)	1.95 (1.40; 2.93)	1.19 (0.89; 1.56)	2.01 (1.58; 2.35)	2.00 (1.66; 2.94)	2.93 (1.66; 4.34)	2.65 (1.93; 3.73)	2.69 (2.09; 3.17)	0.016140 +83.5% *	0.146970 +50.3%	0.003805 +122.7% *	0.057441 +33.8%
FD	0.21 (−0.27; 0.66)	0.68 (0.35; 1.56)	0.20 (−0.11; 0.42)	0.99 (0.48; 1.25)	0.90 (0.58; 1.85)	1.32 (0.72; 1.73)	1.07 (0.73; 1.56)	1.41 (1.01; 2.12)	0.028743 +328.6% *	0.207576 +94%	0.001168 +435% *	0.018225 +42.2% *

Note. *p*—statistical significance (*—*p* < 0.05); 1TCM—one tissue compartment mode; AC—attenuation correction; NAC—non-attenuation correction; FD—flow difference; MBF—myocardial blood flow; MFR—myocardial flow reserve.

**Table 9:** ROC analysis of regional quantitative indices of dynamic SPECT. The «gold standard» is FFR.

Model	AUC	SE	95% CI	COV	Se	Sp	*p* Value
Regional Stress MBF, mL/min/g							
Net Retention, AC	0.82	0.09	0.65–0.98	≤1.24	69.2	90.9	0.0003
Net Retention, NC	0.72	0.094	0.53–0.90	≤1.86	46.2	90.1	0.02
1-Compartment, AC	0.63	0.099	0.43–0.82	≤2.63	84.6	45.5	0.21
1-Compartment, NC	0.64	0.1	0.46–0.84	≤2.21	84.6	50	0.16
Regional CFR							
Net Retention, AC	0.79	0.08	0.92–0.91	≤1.79	76.9	81.8	0.0004
Net Retention, NC	0.68	0.095	0.5–0.83	≤2.35	79.9	59.1	0.06
1-Compartment, AC	0.75	0.09	0.57–0.88	≤1.79	84.6	72.7	0.007
1-Compartment, NC	0.64	0.097	0.46–0.795	≤2.35	69.2	63.6	0.14
Regional DF, mL/min/g							
Net Retention, AC	0.82	0.08	0.64–0.98	≤0.45	84.6	86.3	0.0001
Net Retention, NC	0.72	0.09	0.56–0.899	≤1.54	92.3	50.0	0.0097
1-Compartment, AC	0.72	0.095	0.54–0.91	≤0.66	76.9	72.7	0.018
1-Compartment, NC	0.622	0.1	0.42–0.83	≤0.63	53.8	77.2	0.24

Note. AUC—area under the ROC curve; SE—standard error; 95% CI—95% confidence interval; *p*—significance level (area = 0.5); COV—cut-off value; Se—sensitivity; Sp—specificity.

## 4. Discussion

Assessment of the absolute flow measurement from dynamic SPECT is of considerable importance, since it allows one to overcome the problems related to underestimating myocardial perfusion in patients with severe multivessel atherosclerotic disease [17]. Additionally, dynamic SPECT data can assist in diagnostics, and investigating diseases is associated with microvascular changes [17].

The diagnostic effectiveness and informative nature of this approach for myocardial perfusion evaluation were shown in studies involving the PET approach, since the technology of acquisition and reformatting of dynamic tomographic frames were available only for this method, until recently [1].

Modern advances in SPECT provide quantitative data on myocardial perfusion similar to PET measurements [3,4,18,19]. Despite the similarity of the methods and strong correlation of measurements, certain differences are found. These relate to the technician principles of data acquisition, the pharmacokinetics of used tracers, and post-processing algorithms. This concerns, in particular, the choice of flow kinetic models and providing the feathers–attenuation correction, such as being more useable.

This study is one of the first aimed at comparing the tracer kinetic models for dynamic SPECT and also assessing the effect of attenuation correction on quantitative perfusion indices: MBF and CFR (on clinical data).

The positive findings of our investigation are that both models—1-compartment and net retention—provided results generally close enough to be used interchangeably. Separately, we must emphasize that attenuation correction contributes significantly to the consistency between the models. Despite this, our results demonstrate the possibility to use both models for the evaluation of values in myocardial flow and coronary flow reserve.

The negative findings can be attributed to the significant variability in the values of dynamic SPECT data, depending on the kinetic model. Also, this is equitable for the attenuation correction procedure, which adds intra-model variability to the results.

Models. A generally accepted concept that allows for making decisions about the selection of one model for dynamic SPECT does not exist at this moment. Comparison flow models for quantitative assessment of myocardial blood flow and reserve are a particular interest and may be used for valorization, standardization, and practical application. Despite the fact that a number of papers have described dynamic SPECT results, there is variation in the use of flow models and attenuation correction. This fact was emphasized in one of the latest papers in this field [20]. Our results provide new insight on the dynamic SPECT method, underling that AC provides more accurate values of MBF and MFR, as compared to ICA and FFR, respectively. The NRM or 1CTM flow models with AC can be used interchangeably. These results might improve dynamic SPECT acquisition and processing protocol standardization, providing more robust clinical results.

A large pool of studies are related to the PET approach. In a review, Morris et al. [21] focused on the radioactive indicator retention features and parameters of the PET system; at the same time, kinetic models were not considered. All kinetic models are based on the principle of a measure of the concentration of radiotracers in the blood as a function of time (time–activity curve), which serves as the model’s input function. According to this assumption, we can draw a conclusion about interchangeable models. Particularly, this suggestion was affirmed by Wells et al. [22,23] in an experimental study, showing that the 1-compartment model produces K1 estimates that are almost as accurate and related to the 2-compartment model fits by a simple scaling factor of 0.8. Moreover, this result is comparable to the literature on ejection fractions [23]. The results were provided by Slomka P. et al. [2], and Lortie et al. [24] demonstrated an excellent correlation between the 1-compartment and 2-compartment flow models. Strong correlation between the simple flow model and complete-compartmental model was revealed. Therefore, the authors suggest that both models are acceptable for clinical use. However, in clinical practice, the choice of a certain model depends on technical factors and patient characteristics. On the one hand, the 2-compartment model is more complex and requires high-quality data as well as a high tracer extraction fraction ratio. On the other hand, the use of a simpler model such as 1-compartment or net retention, offers more stable results, with dynamic images of suboptimal quality and less suitable radionuclides, although there may be a loss of accuracy.

However, numerous comparison studies have demonstrated variability in the measurement of myocardial blood flow, depending on the kinetic model [25,26,27,28,29]. Khorsand et al. in 2005 and Nesterov et al. in 2014 [25,26] found different results for PET, depending on the 1- and 2-compartment models. Coxson P. et al. [27] showed that both 1- and 2-compartments models estimate the underlying physiological flow parameters. However, the 1-compartment model is substantially better at differentiating flow in the lower range and is also better in the range of clinical interest. The results of comparative analysis for five different tracer models by Choi et al. [28] also demonstrated various measurements of myocardial blood flow; meanwhile, the correlation between models was strong. A similar result was achieved by DeGrado et al. [29] when the 1-, 2-compartment, and net retention models were compared. In summary, for PET the simplification of the model leads to underestimating the blood flow, increased variability in quantitative values, and most dependent measurements to noise [29,30]. This variability is characterized by a low absolute number range, which means it is not appropriate for clinical practice. However, it leads to a grey-zone appearance, which can harm precise threshold determination and reduce clinical implementation. On the basis that the PET and SPECT approaches are similar, a clinical comparison of the kinetics flow models for dynamic SPECT is currently considered interesting. At present, the method of dynamic SPECT has been shown to reflect states of coronary microcirculation [4,12,17,31,32,33] and, hypothetically, could be used in clinical practice. However, dynamic SPECT could encounter the same complexities as PET: the choice of optimal data collection, post-processing and flow model, and the useability of different correction methods. In the present study, we compared data from the dynamic SPECT, which were obtained for two different models: 1-compartment and net retention in clinical practice. Both models are more acceptable for the SPECT approach, because of the simpler mathematical component appropriated to non-linear indicator retention in the cardiomyocyte [34]. Our results, particularly repeated data of PET research [23,24,25,26,27], demonstrated clinical applicability for these kinetic models, with an underestimation of flow values by the net retention model with attenuation correction. Noticeably, the attenuation correction contributes more to diverse measurement than choosing the kinetic models.

Attenuation correction. Attenuation correction is a procedure that allows for the removal of soft tissue artifacts from SPECT images. The usefulness of this method of correction has been proven in a number of different studies for MPI, and the diagnostic efficiency of its use has been shown [35]. The influence of attenuation correction on the dynamic SPECT results was discussed earlier: Wells et al. [14] showed that motion correction and binding of the tracer to blood improved the accuracy and precision results between dynamic SPECT and PET, but contradictory data were obtained regarding AC, and the authors could not draw an unambiguous conclusion. This assumption is likely related to an insufficient sample size and the experimental study design. This study is more robust in terms of sample size and reference standards. Agostini et al. [4] discussed the need to use AC for the assessment of myocardial blood flow and reserve by dynamic SPECT. The difference in measurements of MBF and CFR by PET and dynamic SPECT could be associated with the use of AC in PET imaging, which was also emphasized. According to Giubbini et al. [3], using attenuation correction increased the correlation between PET and dynamic SPECT but underestimated the flow parameters for the net retention model. Underestimated values of myocardial blood flow were shown by M. Bailly et al. [5] when using the bet retention model with AC. However, the correction did not significantly affect MFR. The clinical value of resting and stress MBF is limited due to the variability and dependence on factors, such as gender, age, and ethnicity. In contrast, MFR is a more stable and relevant index that adequately reflects the state of coronary microcirculation. Therefore, the slight underestimation of blood flow in the net retention model with AC is not clinically significant. However, it should be considered when comparing PET and SPECT indices.

The same findings of an underestimation of flow parameters from the net retention model with AC were obtained in this investigation [3,4,5]. In opposition, increasing values of MBF for the 1-compartment model with AC were found. The results are as follows: for stress MBF, 1.44 mL/min/g (0.89; 2.06) vs. 1.02 mL/min/g (0.67; 1.55); for rest MBF, 0.88 mL/min/g (0.58; 1.31) vs. 0.59 mL/min/g (0.41; 0.77) (for the model with AC and NC). Similar rest and stress myocardial blood flow values for the 1-compartment model with AC were demonstrated by R. Nkoulou et al. and Nesterov et al. [19,26].

In any event, attenuation correction adds significant weight for calculating the quantitative indices of dynamic SPECT. According to the results obtained in this study, including AC in post-processing led to an increasing correlation between models and decreasing repeatability in both models. However intermodal repeatability was strong, independent of AC.

Stress tests (Echo, SPECT, PET, CMR) in the diagnostic algorithm of CAD are used as a method for the identification of ischemia. The anatomical surrogate of ischemia is obstructive atherosclerosis of the coronary artery. Atherosclerosis plaque ≥50% is considered to be one of the most significant criteria of obstructive atherosclerosis. Generally, diagnostic informative dynamic SPECT for the identification of severe atherosclerosis lesions (≥50%) and hemodynamic assessment of plaques for the 1-compartment and net retention models was comparable. The 1-compartment model without AC could be excluded, as it does not allow one to determine the severity of atherosclerotic lesions in the coronary artery. Additionally, the results of comparative ROC analysis indicate higher informative models with AC in the evaluation of significant vessel lesions. It should be noted that stenosis ≥50% does not always lead to impaired microcirculation and, as a consequence, to ischemia [26]. In spite of the fact that the obtained results indicate a greater interrelation of AC models, we cannot fully expound their higher diagnostic accuracy in clinical practice. However, the same pattern for the hemodynamic plaque assessment was not found; more likely, it was related to the insufficient sample size. Also, AUC had high values for all quantification indices of dynamic SPECT—stress MBF, CFR, and DF—for all four models, with the majority of models including AC (non-significant). Despite this, we calculated the cut-off value for dynamic SPECT indices in a similar manner to the one in a previous study using CZT-SPECT [11,32,36] and PET- N13, Rb [36,37].

The quantitative analysis of myocardial perfusion using dynamic SPECT and PET is substantially similar, because it is based on the same principles in its approach. It is important to note that the dynamic SPECT approach can be used in a position PET; the earliest articles on the quantitative evaluation of myocardial perfusion by PET date from the 1990s, with clinical trials in the 2000s; however, clinical standardization and the inclusion of guidelines for this approach were not made available until the present day. There were separate papers, which focused on the technical aspects of acquisition and post-processing data [1,37,38,39]. The focus on a major randomized investigation with the assessment of prognostic data might be able to strengthen PET and dynamic SPECT in clinical practice.

## 5. Conclusions

Dynamic SPECT with the assessment of quantitative flow indices may be considered as an alternative to PET, if PET is unavailable. In some diagnostic scenes, a dynamic SPECT protocol may be reasonable to archive additional diagnostic information, which may influence patient management. The results of our study give rise to the suggestion that the 1-compartment and net retention models correctly reflect the coronary microcirculation and can be used for clinical practice for evaluating quantitative myocardial perfusion by dynamic SPECT. Attenuation correction is an important step in post-processing dynamic SPECT data, which increases the consistency and diagnostic accuracy of models.

## 6. Limitations

The most important limitation of our study relates to the choice of the “gold standard”. ICA is a non-functional approach in the assessment of severity for coronary lesions, and FFR is more appropriate, even if it does not estimate the microcirculation component of the coronary circulation. Moreover, the sample size of the group that provided FFR is not large enough in our opinion. The most adequate method for this kind of investigation is 15O-PET, undoubtedly.

Given that there is a sufficient pool of single-center studies worldwide on the technique of performing and interpreting the results of dynamic SPECT, as well as on the role of this method in different groups of cardiological patients, it is time to conduct a multicenter study focusing on the diagnostic and prognostic capabilities of dynamic SPECT in patients with different cardiological pathologies. Studies should determine the role of this method in cardiological practice.

## Figures and Tables

**Table 1:** Baseline characteristics of included patients.

Parameter	Value
Demographics and clinical profile	
Number of patients	235
Age (years)	59.97 ± 8.82
Male gender, *n* (%)	167 (71%)
Diagnosed coronary artery disease, *n* (%)	174 (74%)
Chest pain, *n* (%)	183 (78%)
Previous myocardial infarction, *n* (%)	61 (26%)
Previous coronary revascularization, *n* (%)	30 (13%)
Family history of coronary artery disease, *n* (%)	157 (66.8%)
Diabetes, *n* (%)	28 (12%)
Hypercholesterolemia, *n* (%)	136 (58%)
Hypertension, *n* (%)	209 (89%)
Current smoking, *n* (%)	94 (40%)
Body mass index (m^2^) (IQR)	28.98 (26.57; 2.08)
Body mass index >30, *n* (%)	101 (43%)
Conventional single-photon emission computed tomography estimates	
Summed stress score	4.0 (2.0; 9.0)
Summed rest scores	1.0 (0.0; 4.0)
Difference between stress and rest score	2.0 (0.0; 4.7)
Ejection fraction (stress), %	66.0 (55.0; 73.0)
Ejection fraction (rest), %	66.0 (57.5; 72.0)
Baseline pharmacotherapy	
Aspirin, *n* (%)	120 (51%)
Beta-blockers, *n* (%)	129 (55%)
ACE inhibitors or ARBs, *n* (%)	127 (54%)
Calcium channel blockers, *n* (%)	73 (31%)
Statins, *n* (%)	176 (75%)
Nitrates, *n* (%)	59 (25%)

Note. ACE—Angiotensin-converting enzyme inhibitors; ARBs—Angiotensin II receptor blockers^;^ IQR—interquartile range.

**Table 2:** ICA and CCTA results.

Parameter	Value
Patient-based analysis (*n* = 235)	
Patients with obstructive (>50%) coronary artery disease, n (%)	235
1-vessel coronary artery disease, *n* (%)	47 (29%)
2-vessel coronary artery disease, *n* (%)	43 (26%)
3-vessel coronary artery disease, *n* (%)	74 (45%)
Vessel-based analysis (*n* = 705)	
Vessel with stenosis >50%, *n* (%)	355 (50%)
Left marginal artery >50%, *n* (%)	12 (3%)
Left marginal artery + left anterior descending artery >50%, *n* (%)	145 (41%)
Circumflex artery >50%, *n* (%)	109 (31%)
Right coronary artery >50%, *n* (%)	101 (28%)
Fractional flow reserve results *(26 patients, 35 vessels)*	
Patients with fractional flow reserve ≤0.8, *n* (%)	13 (50%)
Vessels with fractional flow reserve ≤0.8, *n* (%)	13 (37%)
The average fractional flow reserve value	0.82 ± 0.12
Fractional flow reserve ≤0.8 in left anterior descending artery, *n* (%)	10 (77%)
Fractional flow reserve ≤0.8 in circumflex artery, *n* (%)	1 (8%)
Fractional flow reserve ≤0.8 in right coronary artery, *n* (%)	2 (15%)

**Table 7:** ROC analysis of regional quantitative indices of dynamic SPECT. The «gold standard» is ICA data.

Model	AUC	SE	95% CI	COV	Se	Sp	*p*-Value
Regional Stress MBF, mL/min/g							
Net Retention, AC	0.63	0.030	0.57–0.69	≤2.11	71.2	53.2	<0.0001
Net Retention, NC	0.63	0.032	0.57–0.69	≤2.13	69.9	55.3	<0.0001
1-Compartment, AC	0.64	0.029	0.59–0.70	≤2.00	73.0	50.4	<0.0001
1-Compartment, NC	0.64	0.030	0.58–0.695	≤1.73	61.1	60.7	<0.0001
Regional CFR							
Net Retention, AC	0.63	0.030	0.57–0.69	≤2.11	71.2	53.2	<0.0001
Net Retention, NC	0.63	0.032	0.57–0.69	≤2.13	69.9	55.3	<0.0001
1-Compartment, AC	0.64	0.029	0.59–0.70	≤2.00	73.0	50.4	<0.0001
1-Compartment, NC	0.64	0.030	0.58–0.695	≤1.73	61.1	60.7	<0.0001
Regional DF, mL/min/g							
Net Retention, AC	0.64	0.030	0.58–0.697	≤0.7	76.6	49.2	<0.0001
Net Retention, NC	0.63	0.031	0.57–0.695	≤0.45	55.5	75.2	<0.0001
1-Compartment, AC	0.65	0.029	0.595–0.1	≤0.39	56.6	70.5	<0.0001
1-Compartment, NC	0.65	0.031	0.59–0.71	≤0.23	34.48	88.6	<0.0001

Note. AUC—area under the ROC curve; SE—standard error; 95% CI—95% confidence interval; *p*—significance level (area = 0.5); COV—cut-off value; Se—sensitivity; Sp—specificity.

## Data Availability

The data presented in this study are available on request from the corresponding author. The data are not publicly available due to privacy.

## Supplementary Materials

The following supporting information can be downloaded at: https://www.mdpi.com/article/10.3390/jcm13051271/s1, Table S1: Correlation between models: global stress myocardial blood flow; Table S2: Correlation between models: global rest myocardial blood flow; Table S3: Correlation between models: global myocardial flow reserve; Table S4: Correlation between models: global different flow; Table S5: Association between CA obstruction and CZT SPECT data (logistic regression analysis); Figure S1: Correlation between 1- compartment model with AC and Net Retention model with AC; Figure S2. ROC-analysis of regional quantitative indices of dynamic SPECT; Figure S3. Bland-Altman comparison between models plot.
