# Supplementary material for: The Influence of Kinetic Models and Attenuation Correction on Cadmium–Zinc–Telluride Single-Photon Emission Computed Tomography (CZT SPECT)-Derived Myocardial Blood Flow and Reserve: Correlation with Invasive Angiography Data
Source: J Clin Med. 2024 Feb 23;13(5):1271. doi: 10.3390/jcm13051271 (PMC10932033; doi:10.3390/jcm13051271)
Supplement: Supplementary file 1 [file jcm-13-01271-s001.zip › jcm-2783246-supplementary.pdf]

Supplementary Material - Table S1. Correlation between models: global stress myocardial blood flow

Global Stress MBF	Net Retention, AC	Net Retention, NC	1-Compartment, AC	1-Compartment, NC
Net Retention, AC	1.000	0.788	0.825	0.687
Net Retention, NC	0.788	1.000	0.676	0.793
1-Compartment, AC	0.825	0.676	1.000	0.679
1-Compartment, NC	0.687	0.793	0.679	1.000

Supplementary Material - Table S2. Correlation between models: global rest myocardial blood flow

Global Rest MBF	Net Retention, AC	Net Retention, NC	1-Compartment, AC	1-Compartment, NC
Net Retention, AC	1.000	0.743	0.767	0.621
Net Retention, NC	0.743	1.000	0.609	0.734
1-Compartment, AC	0.767	0.609	1.000	0.624
1-Compartment, NC	0.621	0.734	0.624	1.000

Supplementary Material - Table S3. Correlation between models: global myocardial flow reserve

Global CFR	Net Retention, AC	Net Retention, NC	1-Compartment, AC	1-Compartment, NC
Net Retention, AC	1.000	0.656	0.797	0.548
Net Retention, NC	0.656	1.000	0.551	0.789
1-Compartment, AC	0.797	0.551	1.000	0.547
1-Compartment, NC	0.548	0.789	0.547	1.000

Supplementary Material – Table S4. Correlation between models: global different flow

Global DF	Net Retention, AC	Net Retention, NC	1-Compartment, AC	1-Compartment, NC
Net Retention, AC	1.000	0.694	0.833	0.596
Net Retention, NC	0.694	1.000	0.600	0.817
1-Compartment, AC	0.833	0.600	1.000	0.617
1-Compartment, NC	0.596	0.817	0.617	1.000

Supplementary Material – Table S5. Association between CA obstruction and CZT SPECT data (***logistic regression analysis***)

**Stress MBF**	OR	95% CI	p	AUC,	AUC, p	AUC, 95%CI
ICA						
Patient based (global)						
1TCM_AC	0.62	(0.44-0.87)	0.006	0.63	0.001	0.559-0.697
1TCM_NAC	0.47	(0.29-0.78)	0.003	0.64	0.0007	0.56-0.71
NR_AC	0.49	(0.30-0.78)	0.003	0.64	0.0004	0.57-0.71
NR_NAC	0.70	(0.46-1.07)	0.099	0.57	0.0241	0.52-0.669
Vessel based (regional)						
1TCM_AC	0.73	(0.62-0.85)	<0.001	0.62	<0,0001	0.58-0.66
1TCM_NAC	0.46	(0.35-0.599)	<0.001	0.655	<0,0001	0.61-0.695
NR_AC	0.45	(0.34-0.59)	<0.001	0.65	<0,0001	0.62-0.69
NR_NAC	0.67	(0.53-0.84)	<0.001	0.62	<0,0001	0.575-0.66
FFR (only regional)						
1TCM_AC	0.67	(0.362-1.25)	0.208	0.63	0.20	0.48-0.78
1TCM_NAC	0.57	(0.26-1.24)	0.156	0.64	0.138	0.47-079
NR_AC	0.08	(0.01-0.57)	0.011	0.815	0.0003	0.65-0.925
NR_NAC	0.32	(0.11-0.935)	0.037	0.71	0.02	0.54-0.85

CFR	OR	95% CI	p	AUC,	AUC, p	AUC, 95%CI
ICA						
Patient based (global)						
1TCM_AC	0.64	(0.48-0.85)	0.002	0.65	<0.001	0.58-0.715

1TCM_NAC	0.79	(0.59-1.07)	0.123	0.64	0.001	0.56-0.71
NR_AC	0.755	(0.57-1.0)	0.052	0.59	0.023	0.52-0.62
NR_NAC	0.615	(0.44-0.86)	0.005	0.64	0.001	0.56-0.71
Vessel based (regional)						
1TCM_AC	0.65	(0.55-0.78)	<0.001	0.63	<0,0001	0.59-0.665
1TCM_NAC	0.60	(0.49-0.74)	<0.001	0.64	<0,0001	0.60-0.68
NR_AC	0.67	(0.56-0.798)	<0.001	0.62	<0,0001	0.58-0.655
NR_NAC	0.585	(0.47-0.72)	<0.001	0.64	<0,0001	0.597-0.68
FFR *(only regional)*						
1TCM_AC	0.53	(0.25-1.11)	0.092	0.745	0.01	0.56-087
1TCM_NAC	0.68	(0.42-1.1)	0.118	0.64	0.085	0.499-0.82
NR_AC	0.34	(0.14-0.80)	0.014	0.79	<0.0001	0.66-0.93
NR_NAC	0.54	(0.26-1.14)	0.106	0.67	0.0198	0.54-0.85

DF	OR	95% CI	p	AUC,	AUC, p	AUC, 95%CI
ICA						
Patient based (global)						
1TCM_AC	0.55	(0.37-0.81)	0.003	0.65	0.0002	0.575-0.715
1TCM_NAC	0.52	(0.285-0.94)	0.032	0.63	0.0025	0.55-0.70
NR_AC	0.49	(0.286-0.84)	0.009	0.635	0.0008	0.56-0.70
NR_NAC	0.61	(0.35-1.07)	0.084	0.61	0.01	0.535-0.69
Vessel based (regional)						
1TCM_AC	0.65	(0.54-0.77)	<0.001	0.65	<0,0001	0.61-0.69
1TCM_NAC	0.43	(0.31-0.59)	<0.001	0.67	<0,0001	0.63-0.71
NR_AC	0.46	(0.345-0.62)	<0.001	0.645	<0,0001	0.61-0.68
NR_NAC	0.47	(0.34-0.66)	<0.001	0.64	<0,0001	0.60-0.68
FFR *(only regional)*						
1TCM_AC	0.499	(0.23-1.07)	0.075	0.72	0.0034	0.59-0.89
1TCM_NAC	0.64	(0.28-1.44)	0.276	0.63	0.081	0.49-0.81
NR_AC	0.165	(0.04-0.655)	0.011	0.82	<0,0001	0.695-0.95
NR_NAC	0.41	(0.15-1.11)	0.079	0.73	0.0086	0.56-0.86

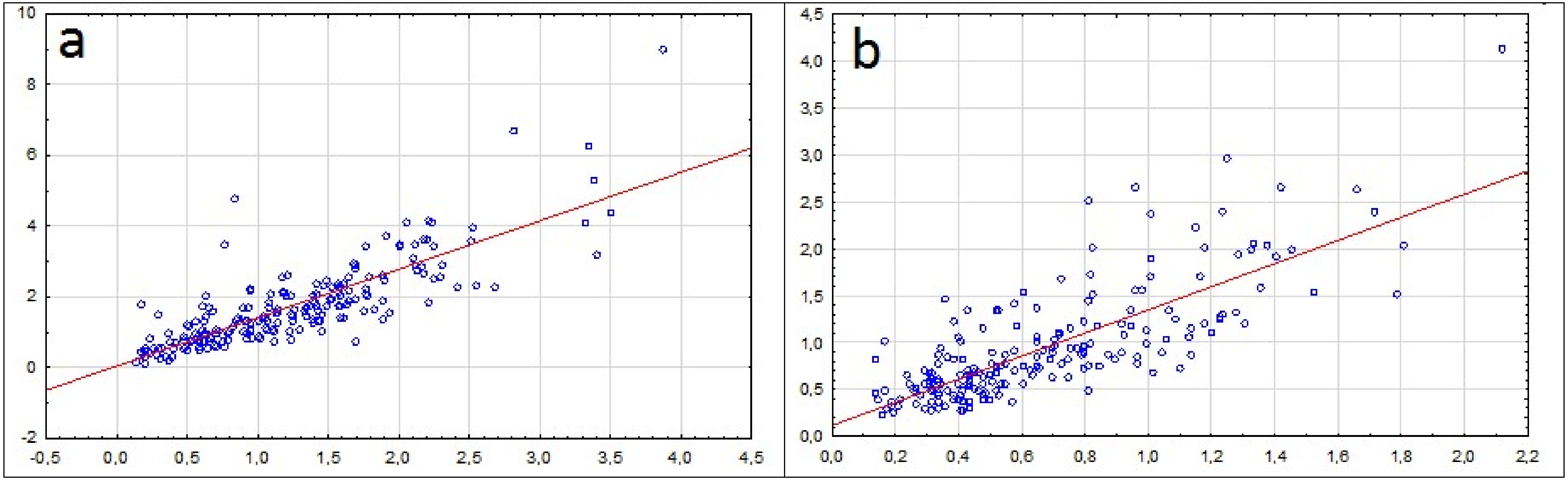
a
10
8
6
4
2
0
-2
-0,5
0,0
0,5
1,0
1,5
2,0
2,5
3,0
3,5
4,0
4,5
b
4,5
4,0
3,5
3,0
2,5
2,0
1,5
1,0
0,5
0,0
0,0
0,2
0,4
0,6
0,8
1,0
1,2
1,4
1,6
1,8
2,0
2,2

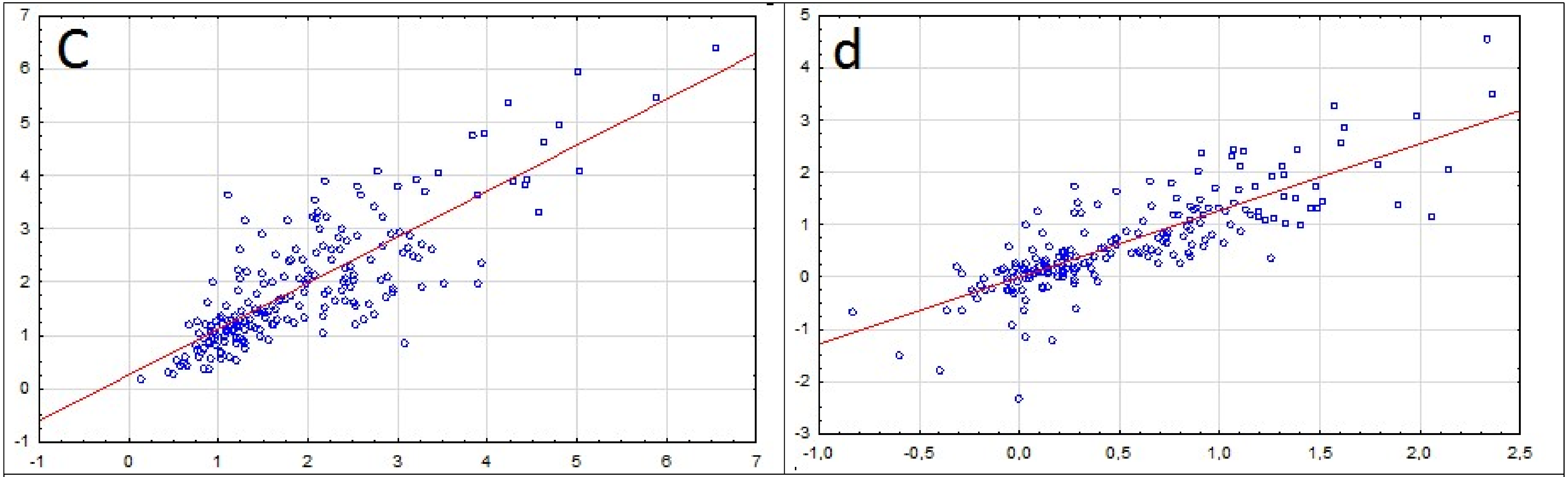

Figure S1. Correlation between 1- compartment model with AC and Net Retention model with AC. a – stress MBF; b – rest MBF; c – MFR; d – FD.

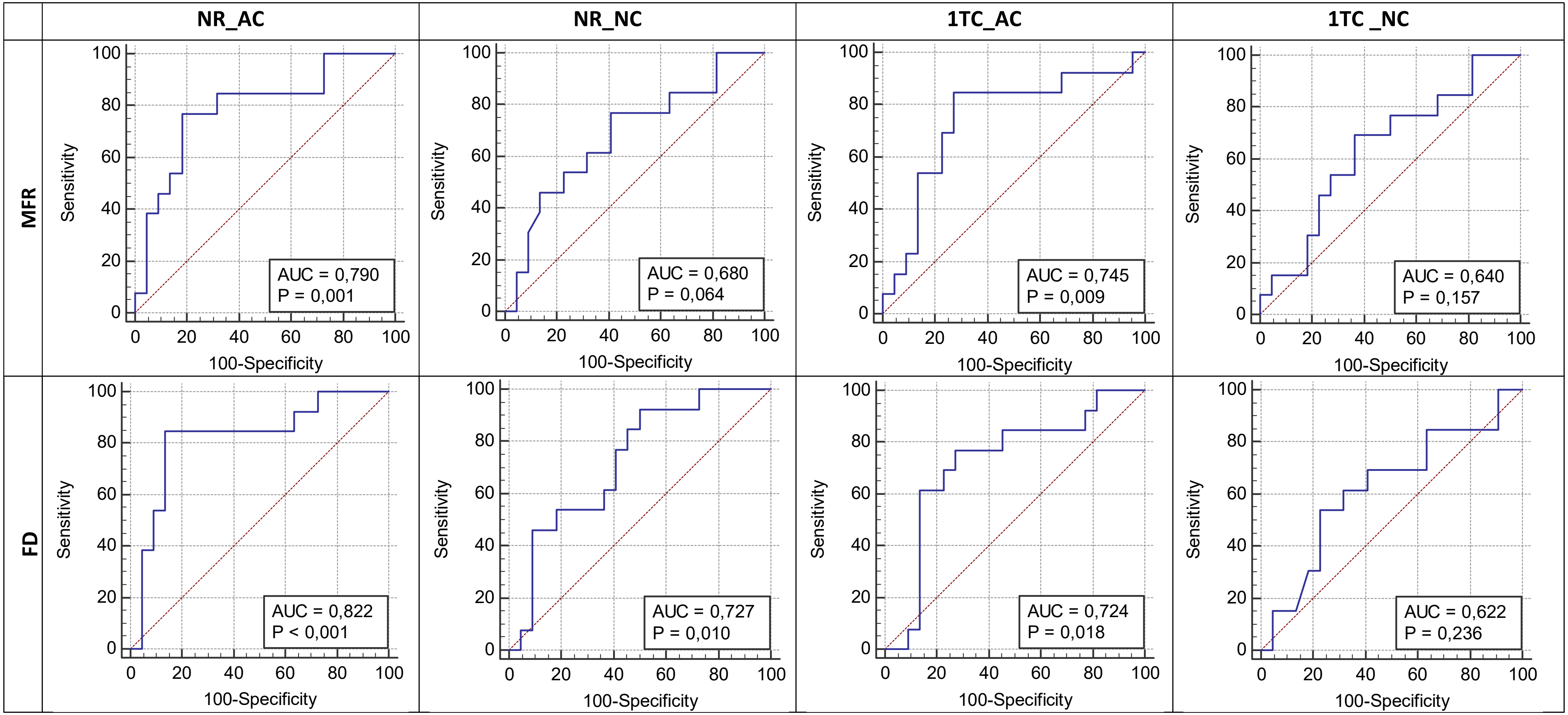
NR_AC
NR_NC
1TC_AC
1TC _NC
MFR
FD
Sensitivity
100-Specificity
AUC = 0,790
P = 0,001
AUC = 0,680
P = 0,064
AUC = 0,745
P = 0,009
AUC = 0,640
P = 0,157
AUC = 0,822
P < 0,001
AUC = 0,727
P = 0,010
AUC = 0,724
P = 0,018
AUC = 0,622
P = 0,236

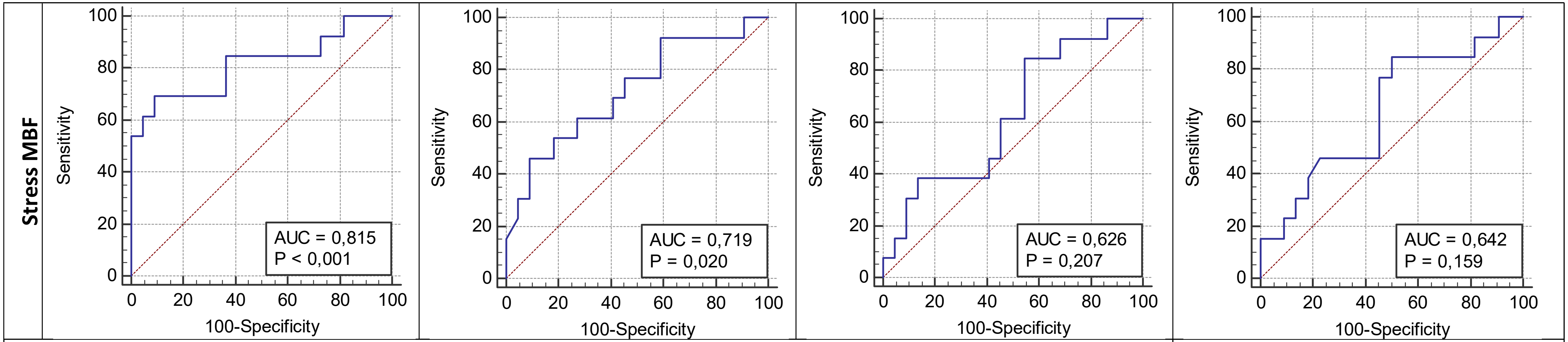

Figure S2. ROC-analysis of regional quantitative indices of dynamic SPECT. The «gold standard» is FFR.

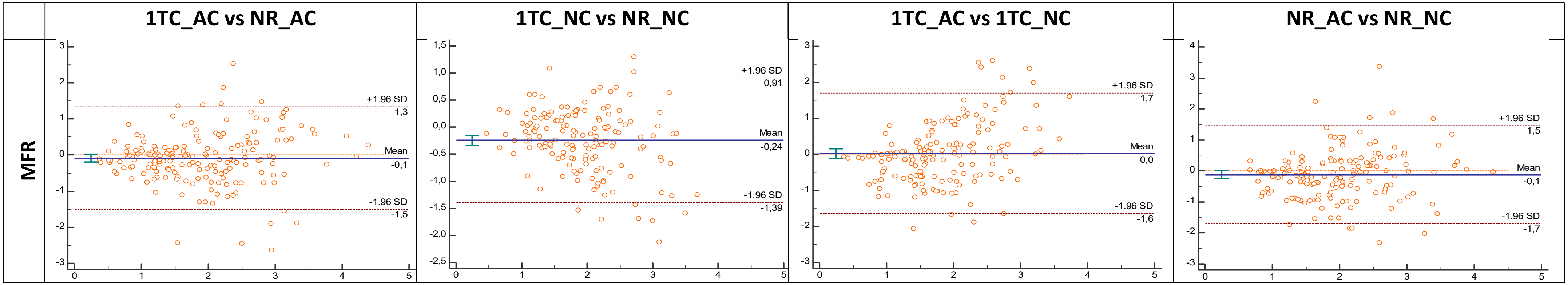

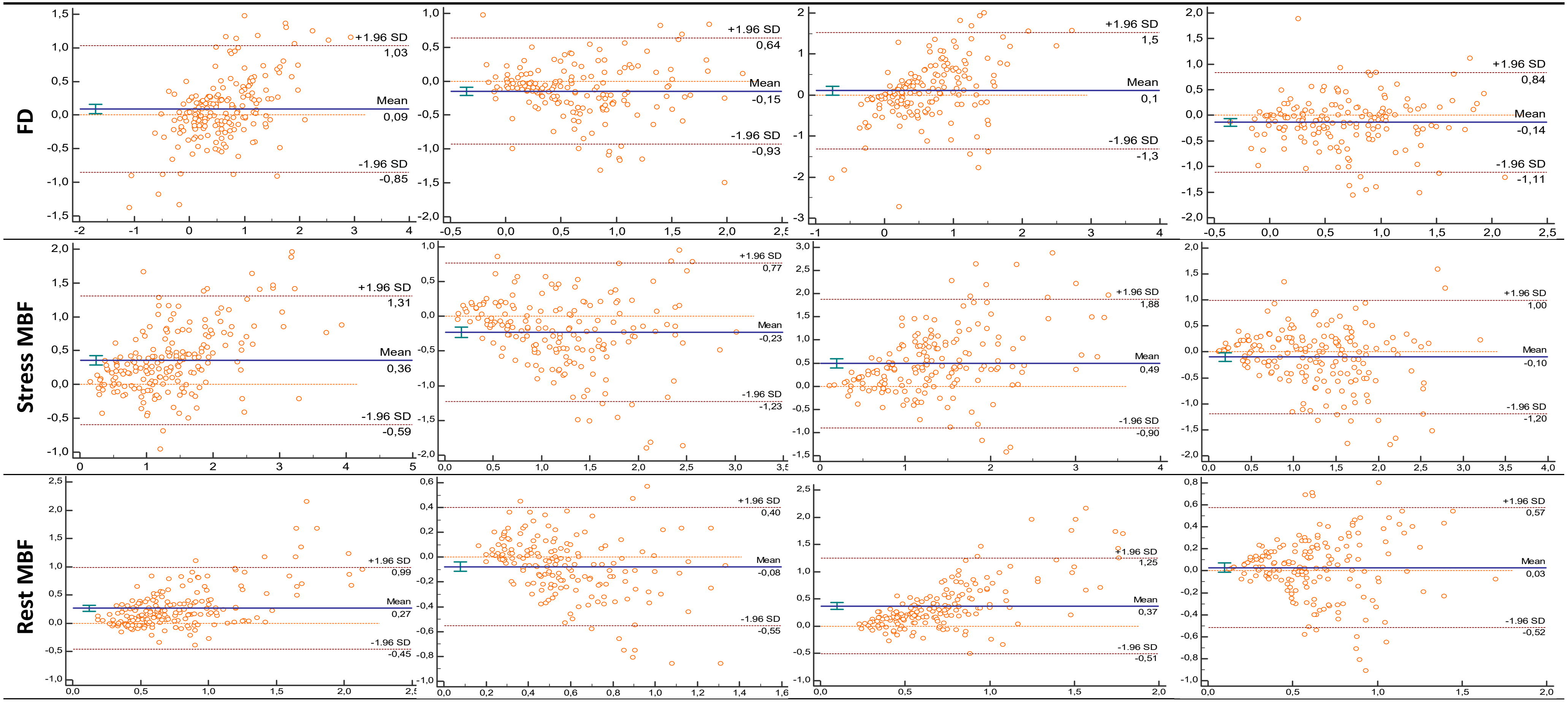

Figure S3. Bland-Altman comparison between models plot.
